# Papillary thyroid carcinoma: an interesting case report

**DOI:** 10.11604/pamj.2025.50.9.45035

**Published:** 2025-01-06

**Authors:** Shivali Kalode, Prajakta Ghewade, Prerna Tekulwar

**Affiliations:** 1 Department of Pathology, Jawaharlal Nehru Medical College, Datta Meghe Institute of Higher Education and Research, Sawangi (Meghe), Wardha, Maharashtra, India

**Keywords:** Papillary, thyroid carcinoma, midline neck swelling, pseudo inclusions, case report

## Abstract

The most common neoplasm is thyroid carcinoma, which can be categorized into papillary, follicular, medullary, undifferentiated, and anaplastic cancers based on histological features (ATC). Papillary thyroid carcinoma accounts for 70-80% of thyroid cancer cases and 1% of all malignancies. This disease entity is notorious for spreading to nearby lymph nodes, especially the cervical lymph nodes, and may manifest as a developing mass on the side of the neck. However, because of the palpable mass, these lesions usually become apparent and are detected quickly. We are reporting a case of an 18-year-old female, who presented with midline neck swelling. Papillary thyroid carcinoma was diagnosed on histopathology. The patient underwent total thyroidectomy with lymphadenectomy followed by therapeutic radioactive iodine ablation and received T4 suppression treatment. The patient was advised to have regular follow-ups with other systemic examinations. Due to various forms of thyroid carcinoma that raise suspicion of a possible familial cancer, early diagnosis, and thorough evaluation are required.

## Introduction

Papillary thyroid carcinoma (PTC) is the most common endocrine malignancy [1]. Of all malignancies, papillary thyroid carcinoma (PTC) accounts for 1% and 70-80% of thyroid cancers [2]. It is classified according to its histological characteristics into papillary cancer (PTC), follicular cancer (FTC), medullary cancer (MTC), and undifferentiated and anaplastic cancer (ATC) [3]. It may develop from thyroid follicular epithelial cells and para-follicular C cells [4]. These lesions usually have a palpable mass, which makes them easy to identify and diagnose. However, many investigations have provided a thorough description of early metastasis to neighboring lymph nodes, which may manifest as a solid or cystic cervical tumor [5]. Papillary thyroid cancer (PTC) comes in a variety of histological morphological forms, but the most common varieties are conventional [6]. Radiation therapy, gamma rays, radioactive iodine (I-131), benign thyroid illness, and a family history of thyroid cancer are risk factors [7]. Papillary thyroid carcinoma (PTC) is more common in women than in males, with two peak incidence periods in the third and seventh decades of life [8]. Due to its slower growth than other carcinomas, PTC has a better prognosis than others, and long-term survival may be anticipated even if distant metastases are detected [9].

## Patient and observation

**Patient information:** an 18-year-old female came to the Outpatient Department of Surgery at Sawangi (Meghe) Wardha with the chief complaint of midline neck swelling gradually developing over the previous four years. The patient has no significant medical history except for a history of trauma 4 years ago. Since then she started noticing swelling for which she did not take any medical treatment as the swelling was slow-growing and painless. According to the patient, the swelling has increased in size recently. There was no other record of either the constitutional symptoms or the prior trauma.

**Figure 1:**
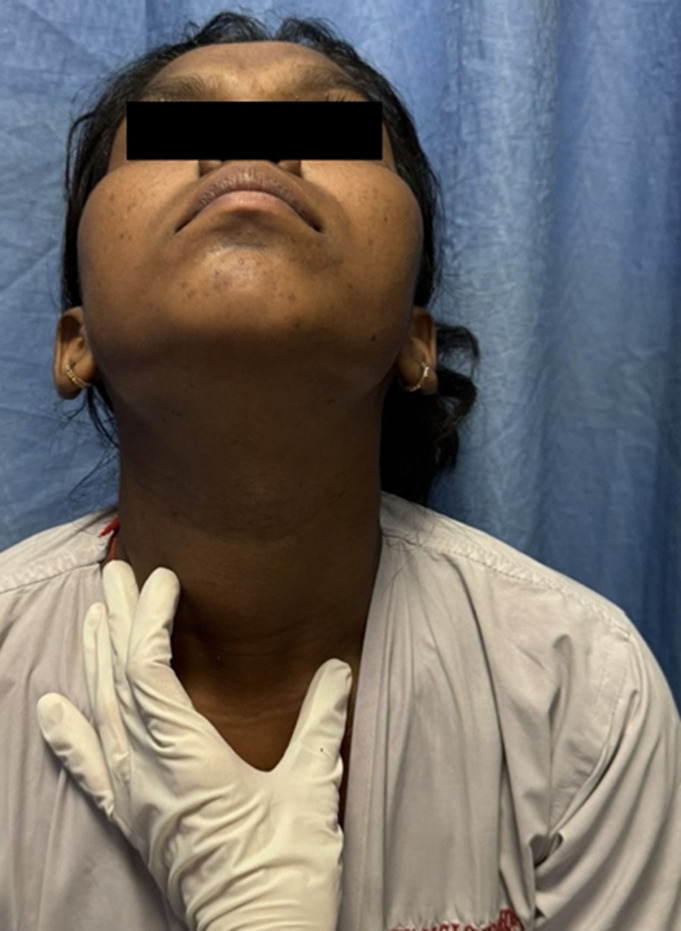
midline neck swelling

**Clinical findings:** on physical examination, all other findings were unremarkable except there was a midline neck mass of size 3x3cm (Figure 1).

**Timeline of the current episode:** midline neck swelling for 4 years, gradually increasing to the present size.

**Figure 2:**
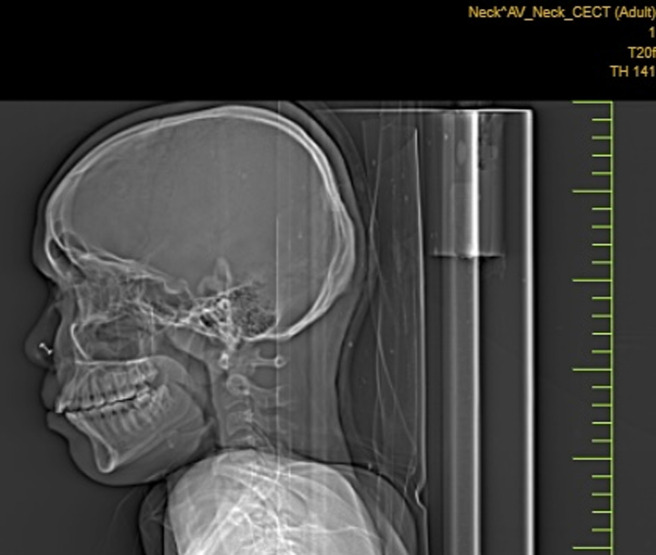
contrast enhanced computed tomography- cystic formation at the midline of the neck

**Figure 3:**
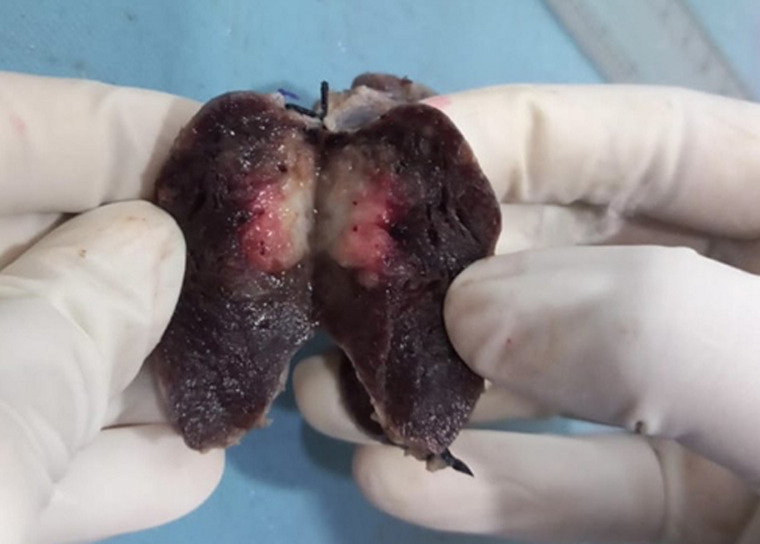
gross image-cut-section of an excised specimen of thyroidectomy

**Figure 4:**
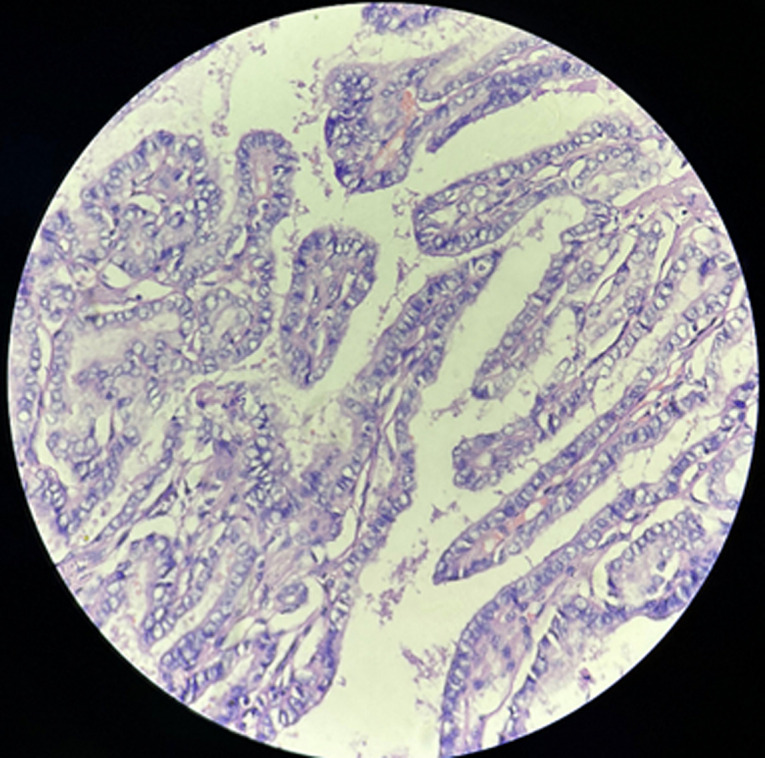
microscopic examination [H&E,40X]- papillary growth pattern with optically clear chromatin and nuclear pseudo inclusions

**Diagnostic assessment:** contrast enhanced computed tomography (CECT) of neck showed cystic formation at the midline of the neck measuring 3x2x1.5 cm (Figure 2). Based on this report the diagnosis of thyroid carcinoma was considered. Following this report, the total thyroidectomy was done and the specimen was sent to the histopathology section in the department of pathology. Grossly, the total thyroidectomy specimen measured 6x4x3cm. A well-defined greyish area on the cut section was soft to firm in consistency measuring 3x2.5x1.8 identified (Figure 3). Microscopic examination at 40X high power view showed a papillary growth pattern with optically clear chromatin and nuclear pseudo inclusions (Figure 4).

**Diagnosis:** histopathological findings confirmed the diagnosis of papillary thyroid carcinoma.

**Therapeutic interventions:** the patient underwent total thyroidectomy with lymphadenectomy followed by therapeutic radioactive iodine ablation and received T4 suppression treatment.

**Follow-up and outcome of interventions:** the patient was advised for regular follow-up with other systemic examinations to rule out recurrence and metastasis.

**Patient perspective:** the patient was satisfied with the diagnosis and treatment.

**Informed consent:** the patient gave written informed consent so that this case report and any related photos could be published.

## Discussion

Papillary thyroid carcinoma, or papillary thyroid carcinoma, is the most prevalent endocrine cancer; it accounts for around 85% of all well-differentiated thyroid malignancies generated from follicular cells with a about 93% 10-year survival rate [1]. Radiation, growth factors, and genetic changes are some of the reasons linked to the development of this tumor. Age, the size of the tumor, and histological characteristics such as lymph node invasion, extracapsular extension, extrathyroidal extension, distant metastasis, and histological variants are all closely correlated with the prognosis of this tumor [2]. According to recent research, the incidence of metastatic illness at the time of diagnosis is close to 50%. There may be almost total cystic degeneration in the affected lymph nodes, making it difficult to distinguish between benign cervical cysts and malignant metastatic illness [5]. In 20% of cases, the tumor is multicentric inside the thyroid gland upon microscopic examination. In 35% of cases, papillary cancer cells' cytoplasm contains round, laminated calcium ions known as psammoma bodies. Unique cytologic and histologic characteristics of papillary carcinoma, such as indentations in the nuclear membrane, cytoplasmic inclusions in the nucleus, and fibrous capsule, enable pathological diagnosis [8].

## Conclusion

The most common form of thyroid cancer is papillary thyroid carcinoma, but it is essential to evaluate thyroid neoplasms in all their forms because of rare forms that vary in morphological aspects, such as areas with cribriform architecture, and follicular, papillary, trabecular, solid, and spindle cell growth patterns with morular areas. In this case, we were able to demonstrate papillary arrangement with optical clear chromatin and nuclear pseudo inclusions which helped to detect and treat papillary thyroid carcinoma. Therefore, solid excrescences and punctate echogenic foci should be specifically identified if a cystic lesion is observed in the thyroid gland to detect potential malignant etiologies such as papillary carcinomas [8].
